# Male median raphe cysts: serial retrospective analysis and histopathological classification

**DOI:** 10.1186/1746-1596-7-121

**Published:** 2012-09-14

**Authors:** I-Hung Shao, Tai-Di Chen, Hsiang-Te Shao, Hsiao-Wen Chen

**Affiliations:** 1 Division of Urology, Department of Surgery, Chang Gung Memorial Hospital and Chang Gung University College of Medicine, No.5, Fusing St., Gueishan Township, Taoyuan County, 333, Taiwan; 2 Department of Pathology, Chang Gung Memorial Hospital and Chang Gung University College of Medicine, No.5, Fusing St., Gueishan Township, Taoyuan County, 333, Taiwan; 3 Division of Dermatology, Department of Internal Medicine, Taipei City Hospital, Renai Branch, No.145, Zhengzhou Rd., Datong Dist., Taipei City, 103, Taiwan

**Keywords:** Median raphe cyst, Paraurethral cyst, Male genitalia cyst, Penile cyst, Genitoperineal cysts, Pathological classification

## Abstract

**Background:**

To review the clinical and pathological characteristics of median raphe cysts and to classify the lesions according to pathogenesis and histopathological findings.

**Methods:**

The medical records of patients who were diagnosed with median raphe cysts between 2001 and 2010 were reviewed to document the clinical presentation and pathological findings of the cysts.

**Results:**

Most patients were asymptomatic; however, 9 patients had inflammatory or infectious cysts that were tender or painful. Four patients who had cysts on the parameatus and distal prepuce had difficulty voiding. Hematuria and hematospermia were noted in 2 cases. Thirty-one cysts were lined with an urothelium-like epithelium, and a squamous epithelium lining was found in 3 cases. In 2 cases, a well-formed mucinous glandular structure was observed. The other 20 cysts consisted of mixed epithelia. After excision of the cysts under local or general anesthesia, an urethral fistula developed as a complication in only 1 case.

**Conclusions:**

Median raphe cysts are benign lesions formed due to tissue trapping during the development of urethral folds. The cysts can be defined into 4 types based on pathological findings: urethral, epidermoid, glandular, and mixed. The associated symptoms and signs should be taken into consideration when determining the treatment for the cysts.

**Virtual slides:**

The virtual slide(s) for this article can be found here: http//http://www.diagnosticpathology.diagnomx.eu/vs/7727074877500751

## Background

**Figure 1:**
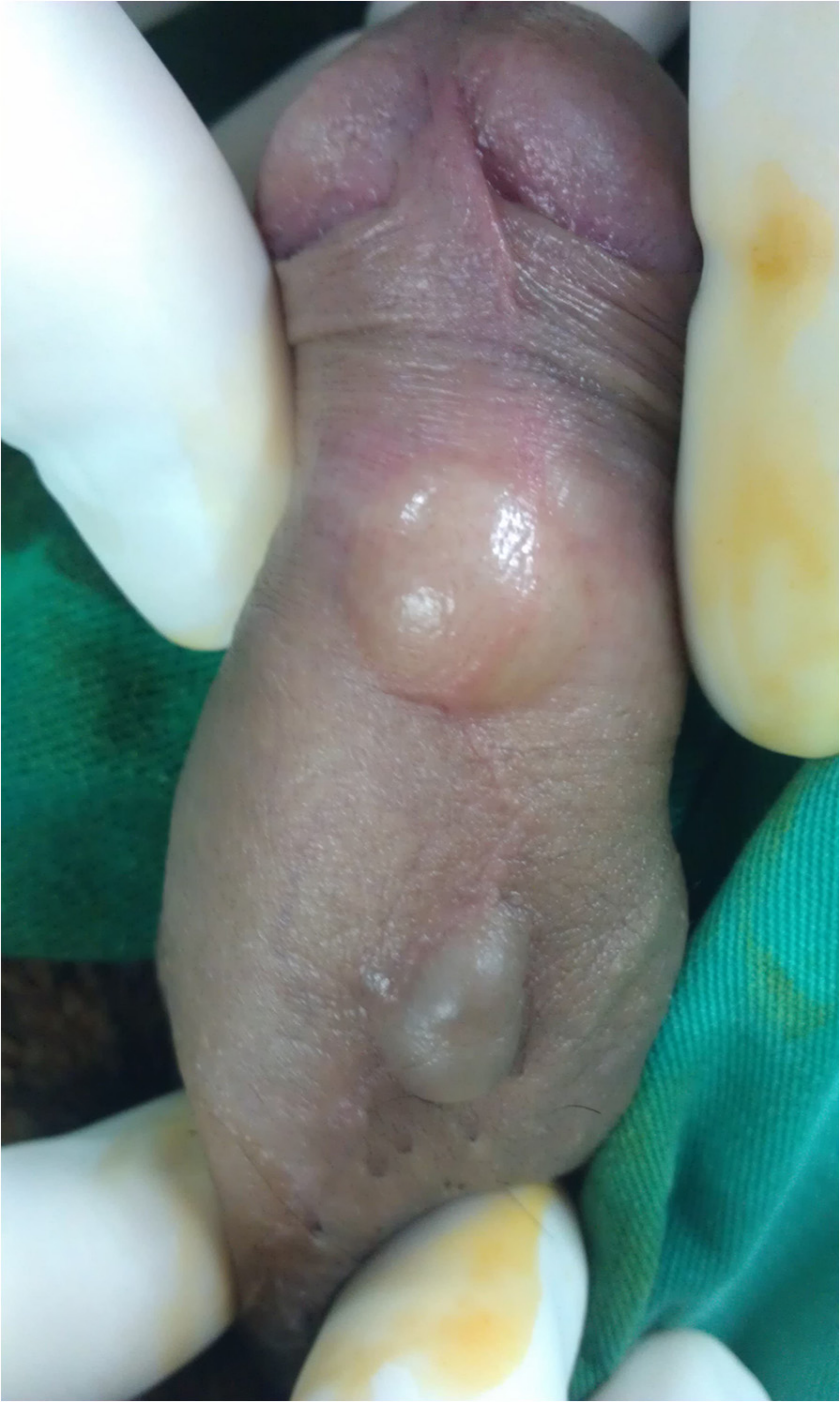
Two synchronous cysts at the ventral side of the penile shaft of a 16-year-old patient.

Median raphe cysts can develop at any site along the midline of the ventral side of the male genital area, from the meatus to the scrotum and perineum (Figure 1). In most patients, the cysts, which are asymptomatic or unrecognized during childhood, may progress later and become symptomatic during adolescence or adulthood. Although these cysts were first identified decades ago, only a few have been reported to date, mostly in case reports. The clinic pathological characteristics and pathogenesis of the disease are not well understood by physicians. In this study, we reviewed the medical records of patients with median raphe cysts to analyze the clinical characteristics and classify the histopathological findings.

## Methods

We retrospectively reviewed the medical records, including the pathological diagnoses, of all patients with cysts on the median raphe (from the meatus to the perineum) diagnosed between 2001 and 2010 in our institution. Patients with epidermal inclusion cysts, skin tags, or cysts located anywhere other than the median raphe were excluded. Medical charts were reviewed to document the clinical presentation, characteristics of the cysts (including the occurrence site, cyst size, and histopathological findings), and patients outcomes.

This study was approved by our institutional review board in Chang Gung Memorial Hospital.

## Results and discussion

**Figure 2:**
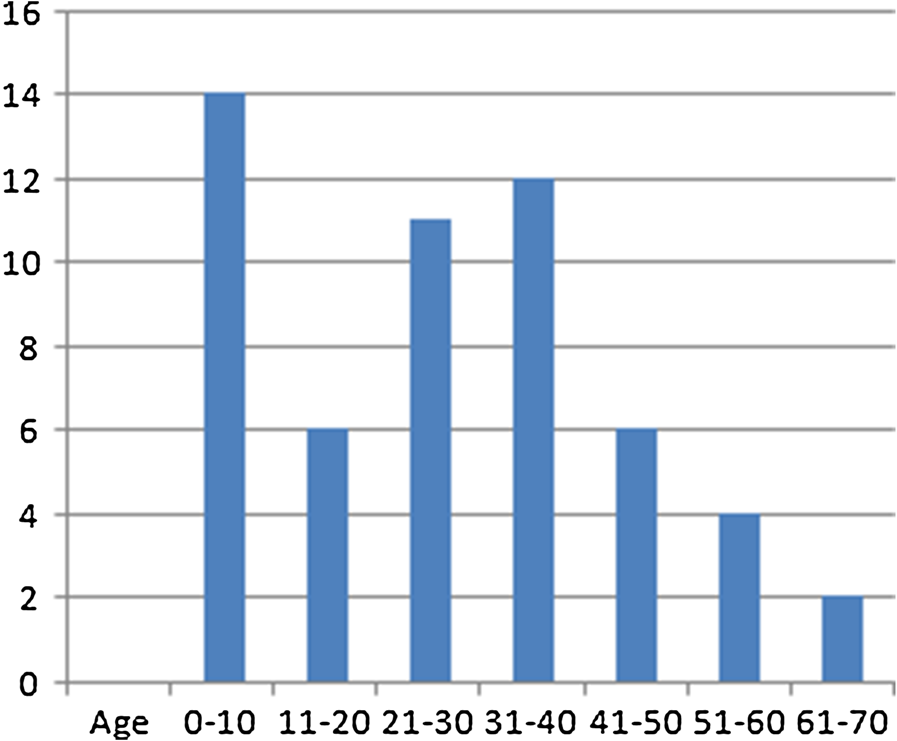
Age distribution with bimodal characteristic.

**Figure 3:**
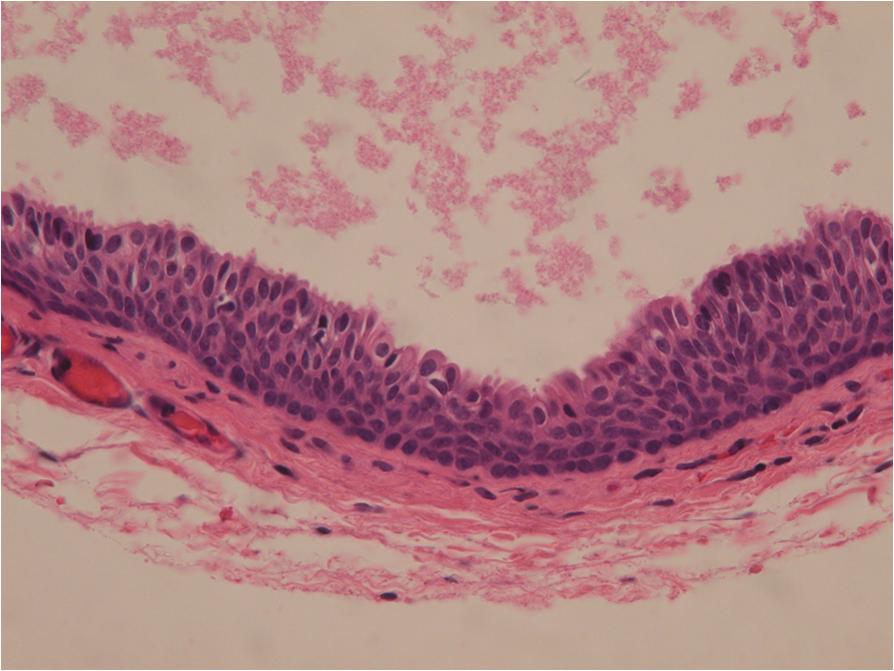
Urethral type epithelium, consisted with one layer of columnar cells overlying varying stratified layers of uniform small cells (Hematoxylin and eosin stain, 400X).

**Figure 4:**
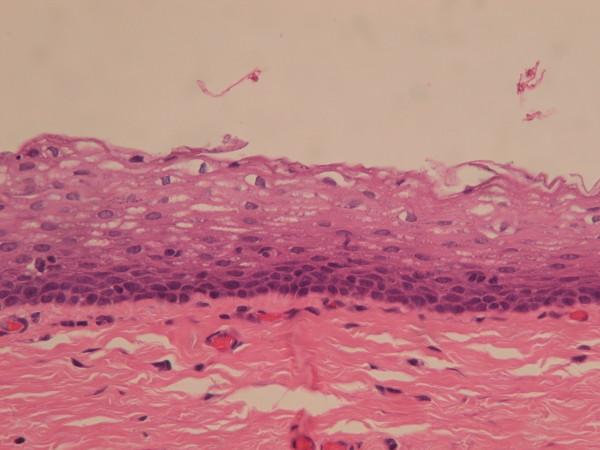
Squamous eputhelium in the median raphe cyst (Hematoxylin and eosin stain, 400X).

**Figure 5:**
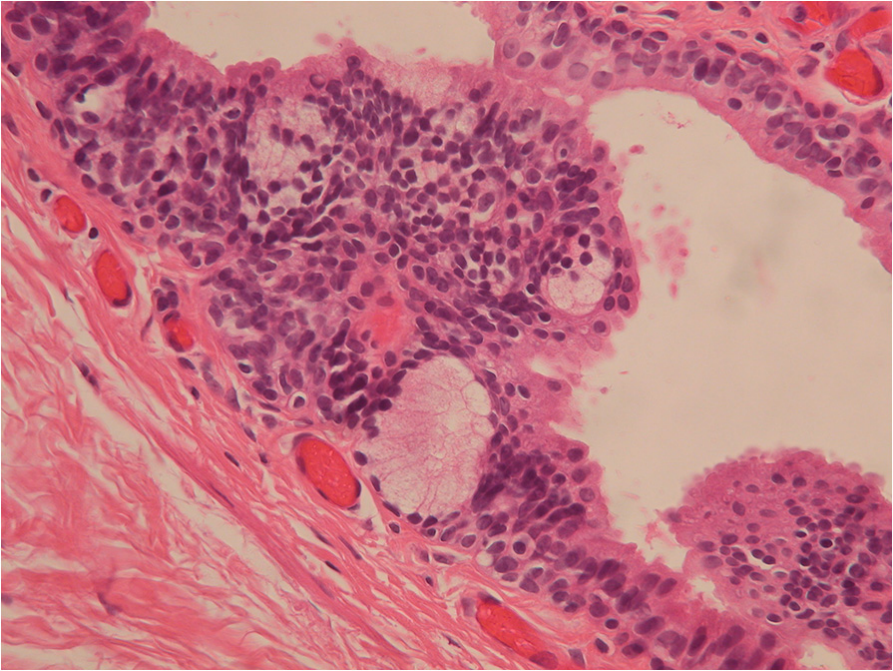
A well-formed intraepithelial glandular structure in the lining of median raphe cyst, resembling Littré gland (Hematoxylin and eosin stain, 400X).

**Figure 6:**
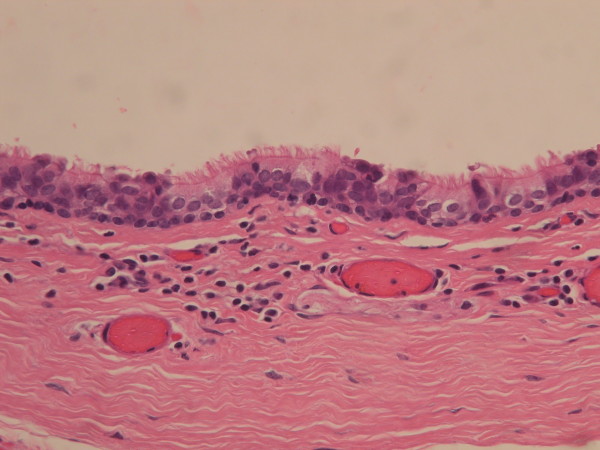
Ciliated cells in median raphe cysts, probably a metaplastic change secondary to local irritation (Hematoxylin and eosin stain, 400X).

A total of 69 genital cysts from 68 patients were reviewed from 2001 to 2010. Ten epidermal inclusion cysts and 3 cystic tumors were excluded. A total of 55 patients (including a 16-year-old patient with synchronous cysts) had 56 median raphe cysts. The clinical characteristics are summarized in Table 1. The mean age of the patients was 26.7 years (range from newborn to 66 years), with a bimodal distribution at approximately 1–10 and 21–40 years old (Figure 2). Most (72.7%) of the patients were asymptomatic, whereas 9 patients (16.4%) had inflammatory or infectious cysts, that were painful or tender. Another 4 patients with cysts on the parameatal site and distal prepuce had difficulty voiding. Hematuria or hematospermia occurred in 2 cases. Cysts were predominantly found on the parameatal site and penile shaft in 19 (33.9%) and 24 cases (42.9%). Lesions were found on the glands penis in 4 cases (7.1%) and on the scrotum and prepuce in 2 (3.6%) and 7 cases (12.7%), respectively. The mean size of the cysts was 0.88 cm, ranging from 0.2 to 2.1 cm. On histopathological examination, all the cysts were centered in the dermis and were not connected with either the epidermis or urethra. They exhibited cystic dilation and were irregularly shaped with clear mucinous content. The histological findings relating to the epithelium lining were varied, as 31 (55.4%) of 56 cysts were lined entirely with a urothelium-like epithelium (Figure 3), which is composed of a layer of columnar cells overlaid with uniform small cells arranged in stratified layers (1 to >10 layers). Apart from the absence of umbrella cells, the histological features of the epithelium were similar to those of the urothelium. A pure squamous epithelium lining was found in 3 cases (5.4%; Figure 4), and a well-formed intraepithelial mucinous glandular structure was found in 2 cases (3.4%; Figure 5). Twenty cases (35.7%) were of the mixed type, that is, the epithelial lining either coexisted with squamous metaplasia or was scattered with mucinous cells. Two cases had focal ciliated cell metaplasia (Figure 6).

**Table 1:** General data and characteristics of patient

**Average age (range)**	**26.7 y/o (1 y/o – 66 y/o)**
No. Age distribution (%)	
0 ~ 10 y/o	14 (25.5)
11 ~ 20 y/o	6 (10.9)
21 ~ 30 y/o	11 (20.0)
31 ~ 40 y/o	12 (21.8)
41 ~ 50 y/o	6 (10.9)
51 ~ 60 y/o	4 (7.3)
61 ~ 70 y/o	2 (3.6)
No. symptom/total No. (%)	
Pain / inflammation	9/55 (16.4)
Difficult voiding	4/55 (7.3)
Hematuria	1/55 (1.8)
Hematospermia	1/55 (1.8)
Asymptomatic	40/55 (72.7)
No. location/total (%)	
Parameatus	19 (33.9)
Glans penis	4 (7.1)
Penile shaft	24 (42.9)
Scrotum	2 (3.6)
Prepuce	7 (12.7)
Average diameter of the cyst in cetimeter (range)	
Parameatus	0.80 (0.2 ~ 3.0)
Glans penis	0.95 (0.6 ~ 11.3)
Penile shaft	0.79 (0.2 ~ 1.5)
Scrotum	2.0 (1.9 ~ 2.1)
Prepuce	0.92 (0.5 ~ 1.2)
No. pathological type (%)	
Urethral type epithelium	31 (55.4)
Epidermoid type epithelium	3 (5.4)
Glandular type epithelium	2 (3.4)
Mixed type epithelium	20 (35.7)
No. complication / recurrence (%)	
Fistula	1 (1.8)
None	54 (98.2)

No surgical complications were encountered, except in 1 case where urethrocutaneous fistula formation occurred owing to long-term chronic inflammation adjacent to the urethra. Among the 55 patients, 1 underwent cyst aspiration, and recurrence was noted several months later. No signs of malignancy were found in any of the patients, either clinically or pathologically.

**Figure 7:**
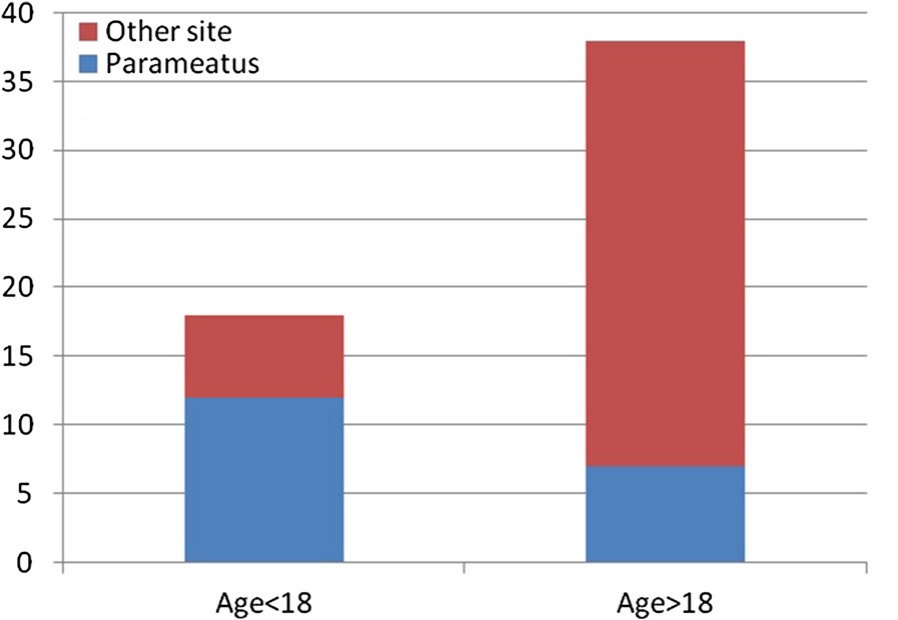
Relationship between the age being diagnosed and the occurrence site.

Median raphe cysts develop along the median raphe of the male external genitalia, and such cysts have been described in case reports; however, serial reports of such cases are rare. In early studies, some cases were reported under varied terms including “mucoid cyst,” “genitoperineal cyst,” “parameatal cyst,” “hydrocystoma,” and “apocrine cystadenoma.”[1] The cysts can occur at any site on the ventral side of the genital area, including the parameatus, glans penis, penile shaft, scrotum, or perineum. Most of the cysts are diagnosed in young patients. Although referred to as median raphe “cysts,” they can also present as a cordlike or canaliform induration on the median raphe. Reports on such cases are even rarer in the literature [2]. The cysts should be differentiated from other genital lesions such as the glomus tumor, dermoid cyst, pilonidal cyst, epidermal inclusion cyst, urethral diverticulum, and steatocystoma [3]. Epidermal inclusion cysts, the most common genital cystic lesion, can occur anywhere on the body in adult men and women. Unlike the median raphe cyst, the epithelium of the epidermal inclusion cyst is lined and filled with keratin. The median raphe cyst can be easily differentiated from other skin tumors by its clinical presentation or pathological features. Median raphe cysts mostly present at birth and may remain asymptomatic or unrecognized during childhood [3]. As the patient grows older, the cyst may also progress slowly. Later in adolescence or adulthood, the cyst may appear as a solitary and movable cystic nodule on the ventral surface of the penis. In some cases, the cyst may progress rapidly or become symptomatic owing to infections or trauma, which could make diagnosis difficult [4,5]. In contrast, some cyts may grow rapidly even in the absence of trauma or infection [6]. However, in most cases, the lesions remain asymptomatic and do not interfere with urinary or sexual function [3]. This could be the reason for the bimodal distribution of the presentation: the patients were either presented to the physicians by their parents during childhood or they visited the physician themselves later in their 20s and 30s because they developed symptoms or mostly for cosmetic reasons. We also found that 12 of 18 cysts were located in the parameatus in the groups younger than 18 years, whereas only 7 of 38 cysts were found on the parameatal site in the group older than 18 years (Figure 7). A possible reason for this is that the cysts on the parameatal site could be easily noticed or were more likely to induce symptoms such as voiding difficulty, prompting parents or patients to seek treatment earlier. The cysts were asymptomatic in 72% of the patients, as most cysts were located on the proximal portion of the genitalia. The more distal the location of the cysts, the greater was the manifestation of symptoms, such as pain or difficulty voiding. Based on the histopathological findings of our serial review, median raphe cysts can be classified into 4 types: urethral, epidermoid, glandular, and mixed. The urethral type is composed of a urothelium-like epithelium, with a layer of columnar cells overlaid with several stratified layers of uniform small cells; this is the most common type, accounting for 55% of all cases. The epidermoid type comprises a squamous epithelium and was noted in 5% of cases. The glandular type, consisting of a well-formed intraepithelial glandular structure in the lining of the urethral epithelium, accounted for 3% of cases. The second most common type, the mixed type, consists of more than 1 type of epithelium, including the urethral epithelium with partial squamous metaplasia, urethral epithelium with scattered or isolated mucinous cells, or all 3 occurring simultaneously, and was found in 35.7% of all cases. Nagore et al [3]. stated that the histomorphological features of the median raphe cyst are related to its embryonic origin and pathogenesis. The urethra forms as a result of fusion of the urethral folds and envelopment of the urethral groove during development. “Tissue trapping” could occur at this time or at a later stage either due to defective fusion of the urethral folds or anomalous outgrowths of the epithelium after primary closure [4]. The fossa navicularis is lined by a non keratinizing squamous epithelium that is similar and continuous to the epithelium covering the glands penis. The histogenesis of this squamous epithelium is explained by either ectodermal in growth or endodermal differentiation [7]. The epithelium of the distal urethra, except the fossa navicularis, is composed of a layer of columnar cells overlying 4–15 stratified layers of uniform small cells, usually categorized as a stratified or pseudo stratified epithelium. Differing from the classical features of the transitional urothelium of the proximal urethra, the nature of the anterior urethral lining appears to be related to that of the squamous epithelium. However, another theory proposed that median raphe cysts could also be caused during development of the ectopic periurethral glands of Littre, which have clear mucinous cytoplasms and basally compressed nuclei resembling pyloric glands of the gastrointestinal tract [8]. This theory explains the presence of intraepithelial mucus cells and glandular structures in some cases. Median raphe cysts are supposed to be formed during the fusion of the urethral folds. Any trapped tissue outside the urethral groove may lead to anomalous outgrowths, thus resulting in the formation of cyst- or canal-like lesions in the median raphe of the genitalia. If the trapped tissue contains urothelium cells, the lining of the formed cyst would contain urethral epithelium. If non keratinizing squamous epithelium cells in the distal urethra are trapped, then we can expect the cyst to consist of an epidermoid epithelium. If the trapped tissue includes the periurethral glands, then the epithelium appears to be glandular. When 2 or more tissues are trapped and develop into outgrowths, cysts with mixed epithelia are formed. Ciliated cells in median raphe cysts are a rare finding, with only 5 cases reported in the English literature. The pathogenesis is probably a metaplastic change secondary to a local irritation [9,10]. Excision followed by primary closure, which establishes hemostasis and prevents infection or cosmetic sequelae, remains the optimal treatment. Given that one of our patients developed a fistula after the operation, excision must be performed with caution when inflammation or adhesion is noted. However, spontaneous regression of the cyst has also been reported, [11] and observation can be considered for those who hesitate to undergo surgery for small and asymptomatic lesions. One patient received aspiration, and recurrence of the cyst was noted a few months later. Thus, simple aspiration alone is not recommended as a treatment for median raphe cysts. Further, marsupialization or unroofing performed in cases of deeply located large cysts, will result in a gaping sinus, which is cosmetically unsatisfactory, and these procedures should be avoided [12].

## Conclusions

The histopathological features of median raphe cysts are varied and can be classified into 4 types of cell manifestations. The diversification of the epithelium may be due to a congenital anomaly of the genitalia, with remnants of embryogenesis or acquired metaplasia of the genitalia. However, no malignant potentiality was observed during long-term follow-up in our study. The associated symptoms and signs should be considered in the treatment of these cysts.

## Competing interests

There are no competing interests in this study.

## Author’s contributions

IHS carried out the preparation of the manuscript draft and participated in the analysis and interpretation of the data. TDC reviewed the pathological results and analyzed and interpreted the data. STS participated in the data collection. HWC participated in the revision and editing of the manuscript. IHS and TDC contributed equally to this study. All the authors read and approved the final manuscript.
